# GRB14: A prognostic biomarker driving tumor progression in gastric cancer through the PI3K/AKT signaling pathway by interacting with COBLL1

**DOI:** 10.1515/biol-2025-1084

**Published:** 2025-04-29

**Authors:** Chun-Bin Gu, Chuang Wang

**Affiliations:** Medical College, Soochow University, Suzhou, 215006, P.R. China; Department of General Surgery, Sheyang County People’s Hospital, Sheyang, 224300, P.R. China; Department of General Surgery, Hulunbuir People’s Hospital Affiliated to Soochow University, Hulunbuir, 021000, P.R. China

**Keywords:** gastric cancer, GRB14, COBLL1, P13K/AKT signaling pathway

## Abstract

Gastric cancer (GC) is a prevalent malignancy with a high incidence rate. Growth factor receptor-bound protein 14 (GRB14) is crucial in cell signal transduction and is associated with tumor growth, invasion, and metastasis. The aim of this study is to investigate the impact of GRB14 on GC growth and metastasis. GRB14 expression and prognosis in GC tissues were analyzed using bioinformatics. The GC cell lines, SGC-7901, MGC-803, BGC-823, and normal gastric epithelial cell line (GES-1) were used in this study. Cell viability, cycle progression, and apoptosis were assessed via CCK-8 and flow cytometry. The colony formation, transwell, and wound-healing assays were conducted to evaluate cell proliferation, invasion, and migration. Protein levels involved in the phosphatidylinositol 3-kinase (PI3K)/protein kinase B (AKT) pathway were analyzed by Western blot. GRB14 expression was significantly higher in GC tissues than adjacent healthy tissues, correlating with poor prognosis. GRB14 knockdown promoted apoptosis and inhibited cell growth, invasion, and migration, while its overexpression exhibited opposite effects. GRB14 directly interacted with cordon-bleu WH2 repeat protein like 1, facilitating PI3K/AKT signaling in GC cells. This study highlights GRB14’s critical role in GC progression and suggests its potential as a therapeutic target.

## 1 Introduction

Gastric cancer (GC) is a malignancy of the gastrointestinal system. It is the prevalent form of malignancy and the second most common cause of cancer-related mortalities globally [1]. Diet, infection, smoking, obesity, and Helicobacter pylori are all associated with GC development [2]. Novel GC cases were reported to be more than 10 million in 2018, with 7.83 million deaths. GC management mainly includes surgery, radiotherapy, targeted therapy, chemotherapy, and immunogene therapy. However, its 5 year survival rate is less than 30% owing to advanced stage, drug resistance, and high recurrence rate [3]. Therefore, creating novel therapeutic targets and finding effective treatments is critical.

Growth factor receptor-bound protein 14 (GRB14) [4] is present in the human genome on chromosome 2. This gene encodes a protein belonging to the molecules vital for regulating cell signaling and growth [5]. The GRB14 protein plays various critical roles within cells. It primarily regulates multiple signaling pathways involving insulin, hormone receptors, and growth factors by interacting with other proteins [6]. GRB14 gene is associated with several diseases and disease-related traits. Reportedly, mutations in the GRB14 gene increased risks of insulin resistance, obesity, and type 2 diabetes [5,7]. GRB14 exhibited abnormal expression in certain tumors and is linked with tumor growth, invasion, and metastasis [8].

The cordon-bleu (COBL) family contributes to morphogenesis and patterning. The COBL WH2 repeat protein like 1 (COBLL1) locus demonstrated a genetic association with the progression of metabolic disorders and the cortical surface [9]. High levels of COBLL1 expression are clinically associated with leukemia. COBLL1 might be involved in the nuclear factor kappa-B (NF-κB) signaling in leukemia cells by stabilizing IKKγ [10]. COBLL1 is androgen-regulated and significantly upregulated in treatment-resistant prostate cancer model cells, where it drives proliferation and migration, highlighting its key role in prostate cancer [11]. Chen et al. [12] illustrated that GRB14 knockout reduced differentiation efficiency, proliferation rate, and lipid storage, while COBLL1 knockout led to excessive lipid storage and lipolysis without impacting adipogenesis. The current study indicated that GRB14 promoted GC progression by positively regulating COBLL1.

The phosphatidylinositol 3-kinase (PI3K)/protein kinase B (AKT) signaling pathway is a well-established signaling pathway inside the cell that has an essential part in modulating cell growth, survival, and metabolic processes [13]. The PI3K/AKT signaling pathway promotes the occurrence and development of GC through participating in epithelial-mesenchymal transition [14]. AKT, a key signaling node downstream of PI3K, is a serine/threonine protein kinase vital for promoting cell survival and regulating multiple functions [15]. Abnormal AKT activity transforms healthy cells into malignant cells. When phosphorylated, AKT enhances the tumor cells’ growth, invasion, and metastasis while suppressing apoptosis in tumor cells [16].

This study explored the association between GRB14 expression, GC clinical characteristics, and outcomes to clarify its underlying molecular mechanisms in GC.

## 2 Materials and methods

### 2.1 Bioinformatics analysis

We conducted a bioinformatics analysis utilizing data from the cancer genome atlas (TCGA) resources (https://portal.gdc.cancer.gov/). RNA-seq data and medical records were extracted. We retrieved data in the format of third-level HTSeq fragments per kilobase of transcript per million mapped reads. A log2 fold change (FC) criterion |FC| > 2 with a detected *p*-value threshold of <0.05 was employed to identify the differential expression genes (DEGs). DEGs from TCGA were identified using gene ontology (GO) and Kyoto encyclopedia of genes and genomes (KEGG) analyses to determine enriched signaling pathways. The clusterProfiler package was utilized to conduct the KEGG enrichment analysis [17]. Employing the TCGA database, we explored the GRB14 content across 370 GC tissues and 32 paired non-cancerous tissues associated with GC tissues. Kaplan-Meier plots were created, and log-rank tests utilizing the package of R survival were conducted for survival analysis. Diagnostic ROC curves were constructed using the pROC package [18].

### 2.2 Clinical specimens

The Institutional Review Board of Hulunbuir People’s Hospital, affiliated with Soochow University, authorized this study. All patients with GC provided informed consent. A total of 25 GC and neighboring healthy tissues were surgically removed and instantly frozen using liquid nitrogen at Hulunbuir People’s Hospital, affiliated with Soochow University.


**Informed consent:** Informed consent has been obtained from all individuals included in this study.
**Ethical approval:** The research related to human use has been complied with all the relevant national regulations, institutional policies and in accordance with the tenets of the Helsinki Declaration, and has been approved by the Institutional Review Board of Hulunbuir People’s Hospital affiliated with Soochow University (Approval No. 2024SYY-08).

### 2.3 Cell culture and reagents

Healthy human gastric epithelial cell line (GES-1) (Catalog No. SNL-304) and human GC cell line (BGC-823) (Catalog No. SNL-140) were procured from Sunncell (Wuhan, China). The human GC cell lines SGC-7901 (Catalog No. TCHu 46) and MGC-803 (Catalog No. TCHu 84) were obtained from the Cell Bank of the Chinese Academy of Sciences (Shanghai, China). The cells were cultivated in RPMI 1640 medium treated with 10% fetal bovine serum (FBS) (Invitrogen; Carlsbad, CA, USA). The medium included 100 µg/mL of streptomycin and 100 U/mL of penicillin (Invitrogen). The cells were cultivated at 37°C, with 5% CO_2_ and 1% O_2_. All experiments were independently conducted in three repetitions. Furthermore, pcDNA3.1/GRB14 and pcDNA3.1/COBLL1 plasmids, shRNA-GRB14, shRNA-COBLL1, and negative control were created chemically by Shanghai Zhongke Biotechnology Co. Ltd (Shanghai, China). Transfection was conducted using Lipofectamine 2000 (Invitrogen, USA) following the manufacturer’s directions [19].

### 2.4 Quantitative real-time fluorescent polymerase chain reaction (qRT-PCR) analysis

Trizol reagent (Life Technologies, USA) was employed to isolate the RNA from freshly frozen tissues or cultivated cells. Beckman-DU800 spectrophotometer was used to determine the absorbance ratios at 260 and 280 nm (A260/280) and assess the purity of nucleic acid. Reverse transcriptase superscript III (Invitrogen, USA) and roughly 2 mg of the overall RNA were used to generate the first-strand cDNA. GRB14 and COBLL1 expression levels were measured in GC tissues or cultivated cells using an ABI Prism 7500 system (Applied Biosystems, USA). GAPDH was used as the internal standard [20] (Table 1).

**Table 1:** Primers used for qRT-PCR analysis of mRNA levels

Target ID	Primer sequence, 5′−3′
GRB14	F: AGGTCGTAGCCGATCGTACG
	R: GAATCGGTACCAATGCAGTAAT
COBLL1	F: ACCTTAAACCGAAGCCTAACC
	R: GAGCAGGTTTCAGAGGACTAAC
GAPDH	F: CCTGCACCACCAACTGCTTA
	R: TCTTCTGGGTGGCAGTGATG

### 2.5 Cell proliferation assay

The cells were transfected and transferred to 96-well plates. Following the manufacturer’s instructions, cell growth was measured daily for 3 days using a CCK-8 assay kit (Beyotime, China). Cell viability was verified by detecting the absorbance at 450 nm utilizing an El×800 instrument (BioTek, USA) [21]. The investigations were performed utilizing a 6× solution and conducted with a minimum of three repetitions.

### 2.6 Cell apoptosis assay

The apoptotic experiments were conducted using annexin V-fluorescein isothiocyanate (V-FITC)/propidium iodide (PI) apoptosis detection kit (BD Pharmingen) following the guidelines of the manufacturer. GC cells were collected in six-well plates at 10^5^ cells/mL density. Annexin V-FITC (5 mL) and PI (5 mL) were introduced into every well. The cells were incubated for 15 min in dark at room temperature and analyzed using flow cytometry (BD LSRII, USA) [22].

### 2.7 Cell invasion assay

The invasive potential of GC cells was assessed employing a Transwell chamber manufactured by Corning Life Sciences. A quantity of 1 × 10^5^ cells was inserted in the top chamber, which was covered with Matrigel, using serum-free media. The bottom chamber was filled with media, including 10% FBS. After a day of incubation at 37°C, the cells on the superior chamber membrane were removed. The cells on the inferior chamber membrane were fixed and treated for 30 min with a 0.1% crystal violet solution and photographed. In contrast to the control, the mean number of invasive cells was quantified as a percentage [23].

### 2.8 Wound healing experiment

A wound-healing assay was used to test cell migration ability. GC cells were placed in a six-well plate and kept at 37°C in an incubator for 24 h. After the cells reached 90% confluence, a line was drawn using a marker on the bottom of the dish, after which a sterile 100 μL pipet tip was used to scratch three separate wounds through the cells, moving perpendicular to the line. The cells were gently rinsed twice with phosphate buffered saline to remove floating cells. Images of the scratches were taken using a microscope (Olympus, Japan) at 0 and 24 h of incubation [24].

### 2.9 Protein–protein interaction (PPI) network creation and functional enrichment analysis

The top 50 genes exhibiting potential PPIs with GRB14 were predicted using the STRING database (https://string-db.org/) [25]. GO and KEGG analyses were performed on the top 50 genes to determine enriched signaling pathways. The clusterProfiler package was used to conduct GO and KEGG [26].

### 2.10 Western blot

Cell lysates were made in radioimmunoprecipitation assay (RIPA) buffer with the addition of a proteinase inhibitor. The cells were broken down, and the protein amount was detected using a colorimetric method and the bicinchoninic acid assay protein assay kit (Beijing Solarbio). Proteins were subjected to separation by sodium dodecyl sulfate polyacrylamide gel electrophoresis and electroblotted onto polyvinylidene fluoride membranes. Subsequently, the membranes were blocked in tris-buffered saline with Tween 20 (TBST) containing 5% BSA for 1 h and then incubated with the primary antibody at 4°C overnight. After being washed three times with TBST (10 min for each wash), the membranes were incubated overnight with the primary antibodies GRB14 (Ag6455, Proteintech), AKT (ab8805, Abcam), p-AKT (66444-1-Ig, Proteintech), COBLL1 (ab272656, Abcam), PI3K (ab140307, Abcam), p-PI3K (20584-1-AP, Proteintech), and GAPDH (sc-47724, Santa Cruz). After incubating for 1 h at room temperature with secondary antibodies, all bands were identified using the Enhanced Chemiluminescence System Kit (MultiSciences in Hangzhou, China) [27].

### 2.11 Colony formation experiment

A six-well plate was used to inculcate the GC cells separately. Following the respective treatments, 500 cells were inoculated per well and underwent 14 days of culturing until a colony was obviously formed; the medium was regularly changed. The colony creation rate was evaluated after methanol fixation and 0.5% crystal violet staining.

### 2.12 Cell cycle analysis

Cell cycle analysis was conducted using flow cytometry. The cells were gathered and kept overnight in 70% ethanol at –20°C following culturing for 1 day in a serum-free medium. Subsequently, the cells were incubated in 500 μL of a pre-prepared PI staining solution at 37°C for 30 min, and subsequently analyzed using a flow cytometer (BD LSRII, USA). The resultant data were analyzed by means of FlowJo software (v10.8.1) [28].

### 2.13 Statistical analysis

The data are presented as mean value ± standard deviation of three distinct investigations. The investigations were conducted independently, with a minimum of three repetitions. The analysis of variance was conducted using the Statistical Package for the Social Sciences software (version 19.0; IBM Corporation, Armonk, NY, USA). Furthermore, the *p* < 0.05 difference was deemed statistically significant.

## 3 Results

### 3.1 GRB14 was upregulated in GC and may have a role as a reliable biomarker for GC prognosis and diagnosis

**Figure 1:**
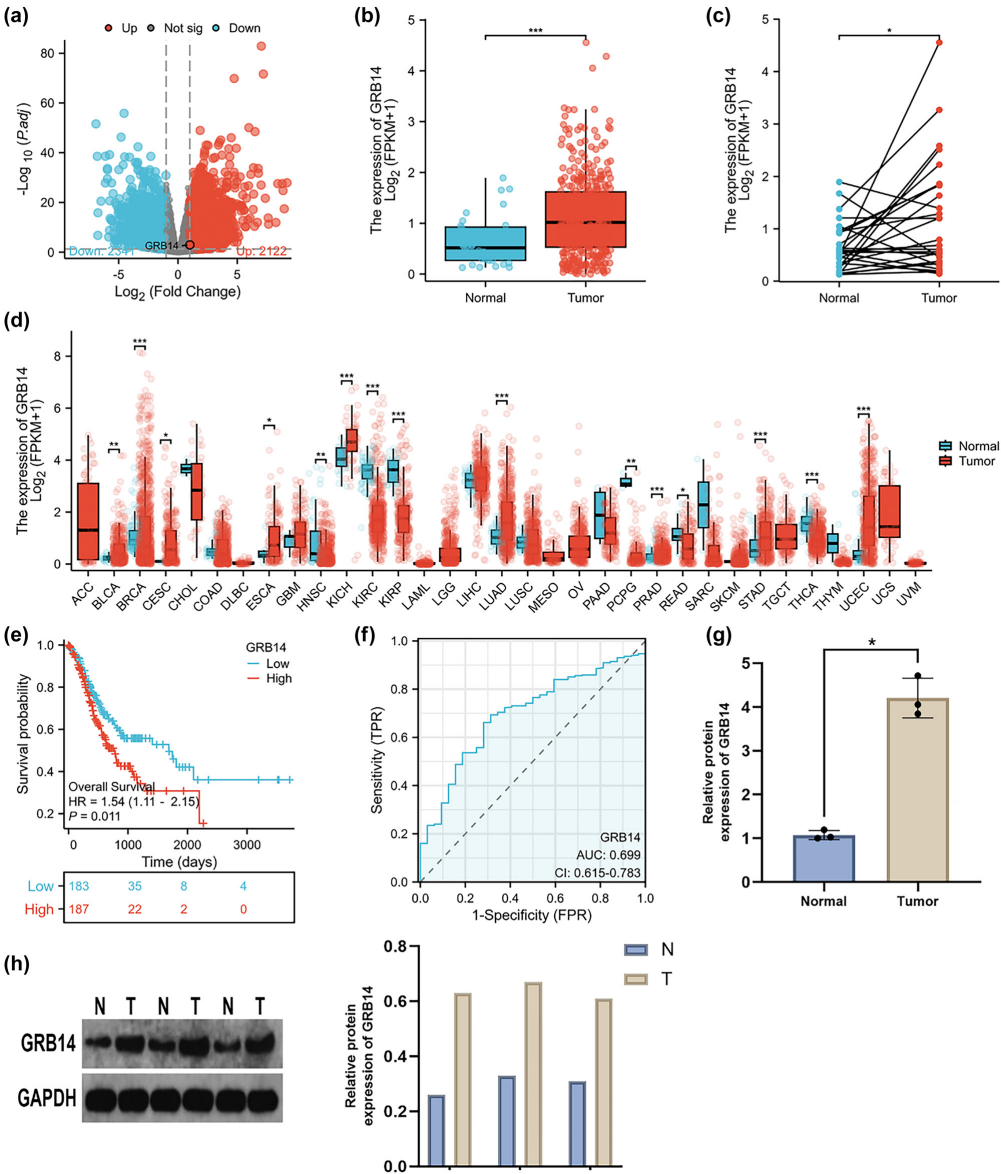
GRB14 Expression and its predictive diagnostic value in individuals with GC from TCGA. (a) Volcano plot of the DEGs. The significant upregulation and downregulation of genes in GC is represented by the red and blue dots. (b and c) Differential expression analysis comparing GRB14 mRNA levels between GC and nearby non-malignant tissues revealed significantly higher levels of GRB14 in GC. (d) Expression levels of GRB14 across different cancers. (e) OS curve for individuals with GC and high (red) and low (blue) levels of GRB14 content. *p* = 0.011. (f) Diagnostic ROC curve for distinguishing GC tissues from normal tissues. (g and h) A comparison of GRB14 levels between GC and nearby healthy tissues was performed using qRT-PCR and Western blot. *p < 0.05; **p < 0.01; ***p < 0.001.

We obtained GRB14 from DEGs for additional analysis. GRB14 mRNA levels were significantly increased in GC tissue than in the nearby healthy tissue (Figure 1a–c). The GRB14 expression pattern was analyzed in various malignancies using the TCGA database. Most cancers exhibited a significant increase in GRB14 levels compared to their respective control tissues (Figure 1d). Kaplan–Meier survival analysis revealed a lesser overall survival (OS) in individuals with GC and increased GRB14 levels (Figure 1e). The predictive efficiency of GRB14 for GC was evaluated using ROC analysis, with an estimated AUC of 0.699 (Figure 1f). Subsequently, GRB14 levels were assessed in GC samples. The qRT-PCR and Western blot outcomes demonstrated a significant rise in GRB14 levels in tumor tissue compared to non-cancerous tissue (Figure 1g and h). These findings indicated a significant increase in GRB14 levels in GC tissue, demonstrating its association with GC progression.

### 3.2 GRB14 promoted cell growth in GC cells

**Figure 2:**
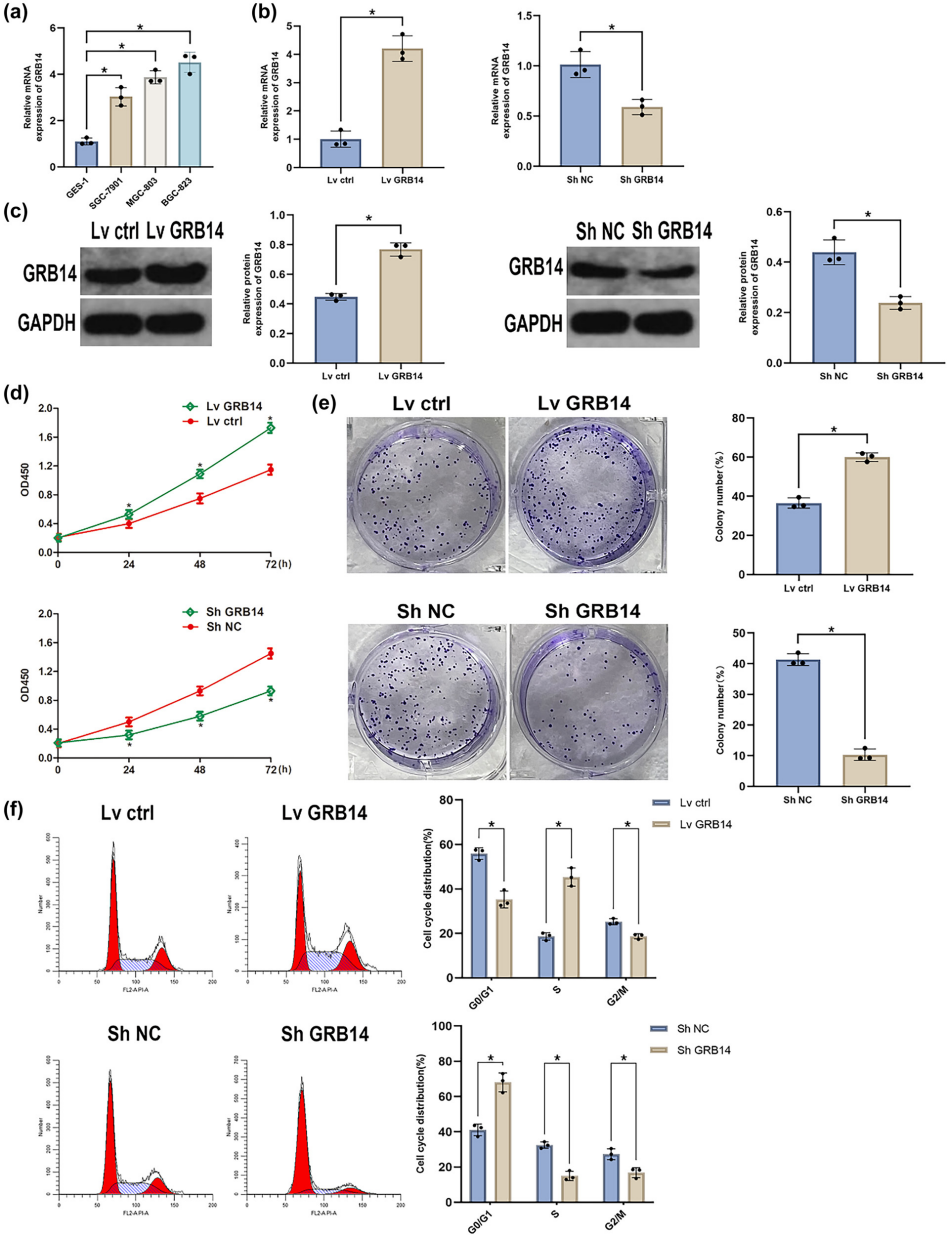
GRB14 enhances cell growth and cell cycle progression *in vitro*. (a) GRB14 levels in GES-1, MGC-803, SGC-7901, and BGC-823 cell lines. (b and c) After transfection, Western blot analysis and qRT-PCR were conducted to assess GRB14 expression levels in BGC-823 cells. (d) Cell viability was assessed using CCK-8 assay at 24, 48, 72, and 96 h post-transfection. (e) Representative images of colony formation assay. (f) Cell cycle analysis by flow cytometry. *p < 0.05.

The GRB14 levels were determined in SGC-7901, MGC-803, BGC-823, and GES-1. The outcomes revealed elevated levels of GRB14 in all GC cells, particularly in BGC-823 cells (Figure 2a). To verify GRB14 function in GC, we transfected BGC-823 cells with sh-RNA targeting GRB14 or plasmids overexpressing GRB14 (Figure 2b and c). CCK-8 assay revealed that GRB14 overexpression significantly enhanced the cell viability while inhibiting GRB14 expression substantially decreased the cell viability (Figure 2d). Colony creation experiments exposed that GRB14 overexpression promoted the growth of individual GC cells, whereas suppressing GRB14 expression suppressed their proliferation (Figure 2e). Cell cycle analysis revealed that GRB14 overexpression significantly increased cell proliferation capacity, whereas GRB14 knockdown noticeably reduced the cell growth capacity (Figure 2f). Our results suggested that GRB14 plays a pivotal function in enhancing the growth of GC cells.

### 3.3 GRB14 exerted anti-apoptotic effects and promoted the invasiveness and migratory capabilities of GC cells

**Figure 3:**
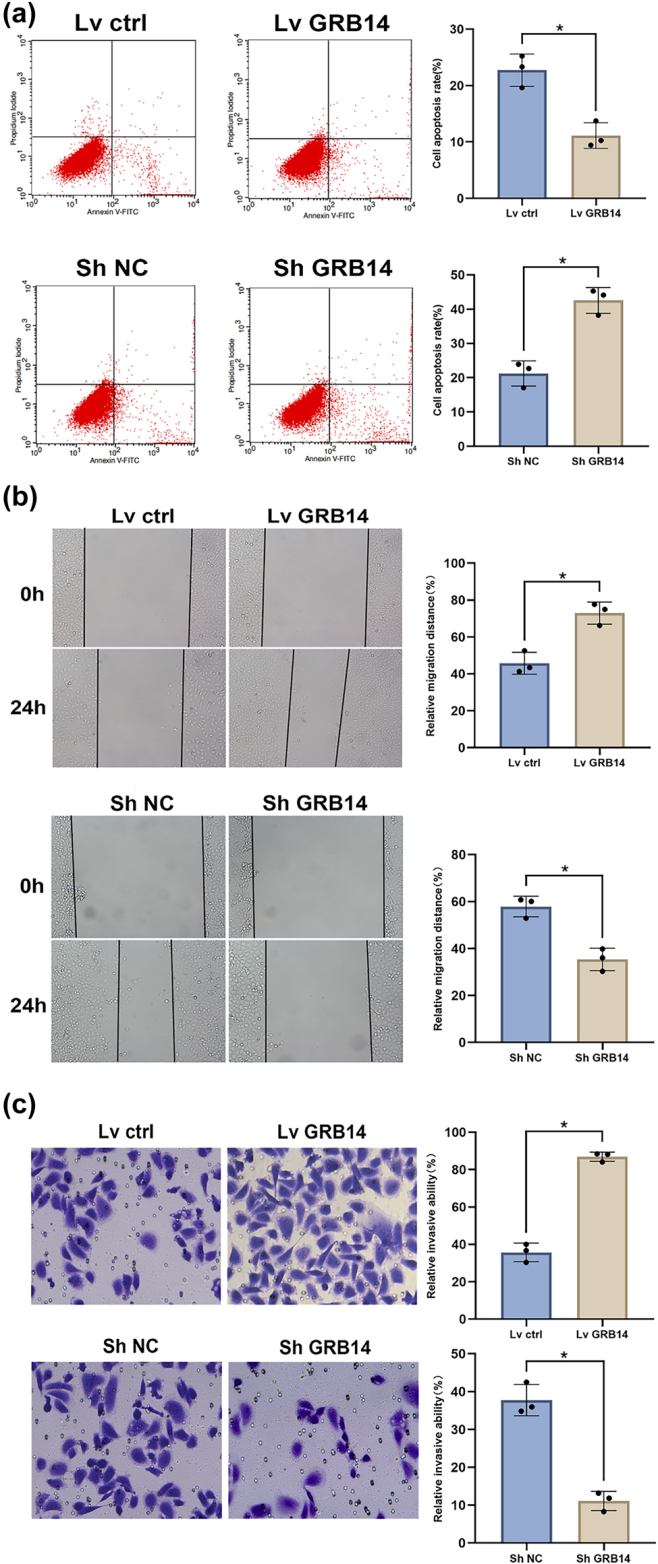
GRB14 exerts anti-apoptotic effects and promotes the invasiveness and migratory capabilities of GC cells. (a) Apoptosis rate measured by flow cytometry in BGC-823 cells. (b) Wound healing evaluation of migration capability in BGC-823 cells. (c) Transwell invasion assay evaluating the BGC-823 cells invasive capability. *p < 0.05.

Next GRB14’s impact on the apoptotic, invasive, and migratory abilities of BGC-823 cells was analyzed. Flow cytometry data indicated that GRB14 overexpression markedly reduced the number of apoptotic nuclei, and inhibiting GRB14 expression reversed the count of apoptotic nuclei (Figure 3a). In wound healing experiments, overexpressing GRB14 significantly increased the migration of GC cells, whereas GRB14 knockdown suppressed their migration (Figure 3b). Transwell assays demonstrated that overexpressing GRB14 promoted cell invasion while silencing GRB14 inhibited cell invasion (Figure 3c). These findings support the hypothesis that GRB14 exerts anti-apoptotic effects and promotes GC cell migration and invasion ability.

### 3.4 GRB14 was greatly connected with the PI3K/AKT signaling pathway

**Figure 4:**
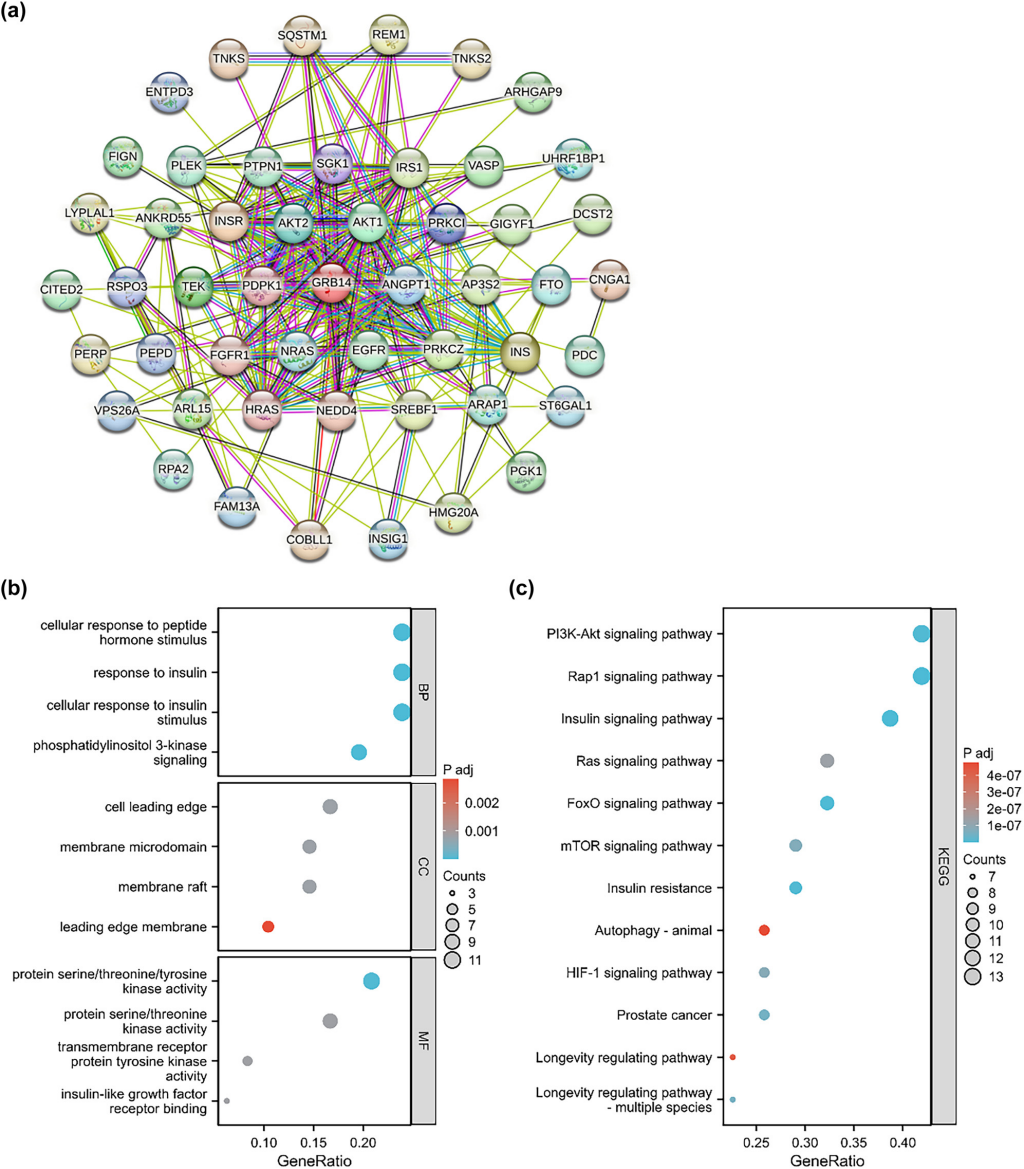
PPI construction and functional enrichment analysis suggested that GRB14 is highly linked to the PI3K/AKT signaling pathway. (a) The top 50 genes associated with GRB14 were detected to create the PPI network. (b) GO enrichment analysis of genes within the PPI network. (c) KEGG enrichment analysis of genes within the PPI network.

We explored the biological GRB14 functions. The STRING database was used to find the top 50 genes most associated with GRB14. A PPI network was generated using the STRING database and Cytoscape software (Figure 4a). GO and KEGG enrichment analyses were conducted on the genes most correlated with GRB14 (Figure 4b and c). The GO analysis revealed the GRB14 connection with the cellular responses to peptide hormone stimulation, insulin and insulin stimulation, and the PI3K signaling pathway. KEGG analysis demonstrated that GRB14 plays a significant function in the PI3K/AKT, Rap1, and insulin signaling pathways.

### 3.5 GRB14 regulated COBLL1 expression in GC cells

**Figure 5:**
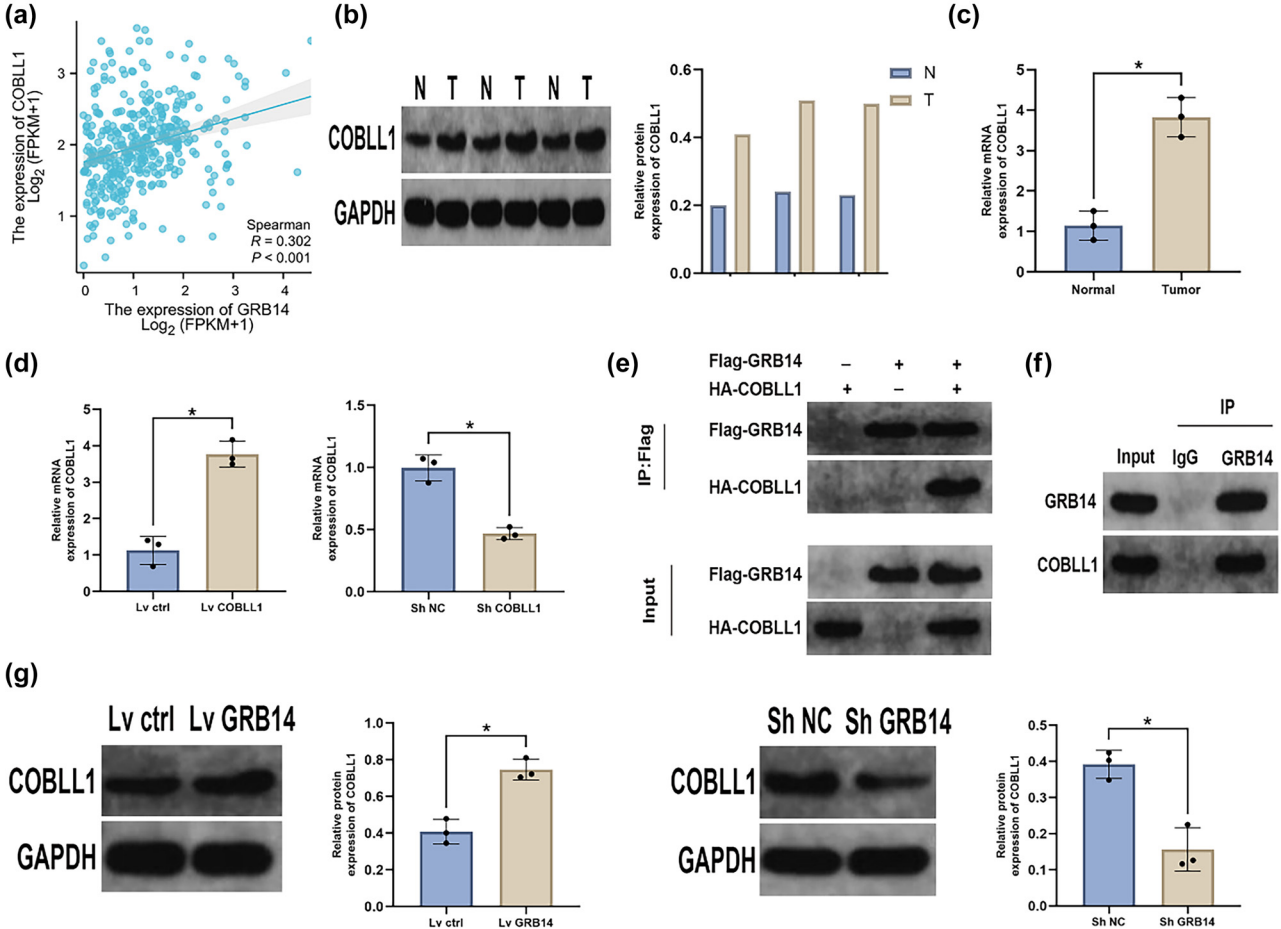
COBLL1 expression in GC cells is modulated by GRB14. (a) COBLL1 expression levels exhibited a robust positive connection with the GRB14 expression levels. (b) and (c) Western blot and qRT-PCR techniques were used to evaluate and quantify COBLL1 expression levels in GC samples and their corresponding adjacent normal tissues. (d) The validation of COBLL1 overexpression and knockdown was performed utilizing qRT-PCR analysis. (e) The Co-IP technique was used to investigate the PPI between exogenously expressed GRB14 and COBLL1 in HEK293T cells transfected with the specified constructs. (f) The validation of GRB14 and COBLL1 interactions in BGC-823 cells was conducted using endogenous Co-IP assays. (g) The COBLL1 expression in BGC-823 cells was evaluated by Western blot analysis after manipulating GRB14 through knockdown or overexpression. *p < 0.05.

This study found a significant positive link between GRB14 and COBLL1 in GC (Figure 5a). Western blot and qRT-PCR results indicated a significant increase in COBLL1 levels in tumor tissue compared to non-cancerous tissue (Figure 5b and c). To verify COBLL1 function in GC, we transfected BGC-823 cells with sh-RNA targeting COBLL1 or plasmids overexpressing COBLL1 (Figure 5d). We conducted exogenous and endogenous Co-Immunoprecipitation (Co-IP) assays in HEK293T and BGC-823 cells, respectively, to validate the direct interaction between GRB14 and COBLL1 (Figure 5e and f). The Western blot analysis revealed a direct relationship between high GRB14 expression and COBLL1 levels in BGC-823 cells. In contrast, the decrease in COBLL1 expression was associated with the inhibition of GRB14 in BGC-823 cells (Figure 5g).

### 3.6 GRB14 modulated GC cell growth, invasion, and PI3K/AKT signaling pathway activation via interacting with COBLL1

**Figure 6:**
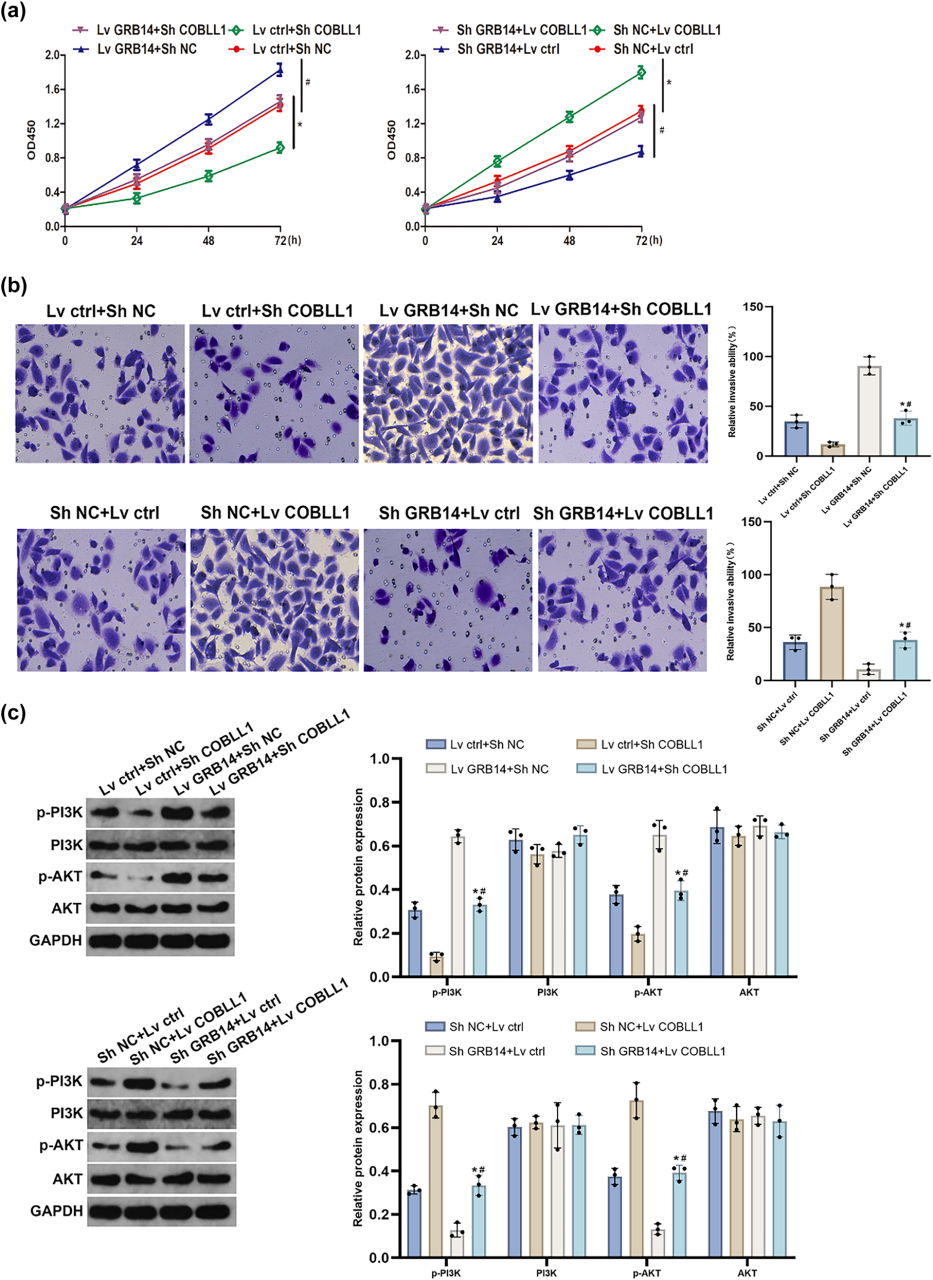
GRB14 modulates GC cell growth, invasion, and activation of the PI3K/AKT signaling pathway by interacting with COBLL1. (a) The BGC-823 cell growth was evaluated using the CCK-8 assay. (b) The invasive potential of BGC-823 cells was assessed using a Transwell invasion assay. (c) PI3K, p-PI3K, AKT, and p-AKT protein expression levels in BGC-823 cells were assessed using Western blot analysis. *p < 0.05 vs Lv ctrl + Sh COBLL1 or Sh NC + Lv COBLL1 group; ^#^ *p* < 0.05 vs Lv GRB14 + Sh NC or Sh GRB14 + Lv ctrl group.

CCK-8 assay exposed that the upregulation of GRB14 significantly enhanced cell growth, which was subsequently counteracted by COBLL1 downregulation. Conversely, GRB14 downregulation notably decreased cell proliferation, which was reversed by the upregulation of COBLL1 (Figure 6a). The results of transwell assays revealed that inhibition of COBLL1 restored the enhanced cell invasion induced by upregulation of GRB14, while overexpression of COBLL1 reinstated the diminished cell invasion resulting from suppressing GRB14 (Figure 6b). The Western blot analysis indicated that COBLL1 suppression restored PI3K and AKT phosphorylation, which were initially enhanced by GRB14 overexpression. Conversely, the upregulation of COBLL1 recovered p-PI3K and p-AKT expression levels, which were suppressed upon silencing GRB14 (Figure 6c). Our results indicated that GRB14-mediated upregulation of COBLL1 expression promoted proliferation, invasion, and PI3K/AKT signaling pathway activation in GC cells.

## 4 Discussion

GC ranks among the most lethal cancers worldwide [29]. The 5 year survival rate following surgical treatment for cancer of the gastrointestinal tract in the early stage can go above 91% [30]. However, most patients with GC are diagnosed at advanced to later stages or when the disease has metastasized, eliminating the optimal treatment window and making the cure challenging [31]. GC progression is influenced by numerous factors like diet, environment, and genetics [32]. There is a close association between the incidence and development of GC and certain molecular biomarkers [33]. These molecules serve as early diagnostic indicators for GC and potential therapeutic targets. The investigation and identification of novel molecular biomarkers in GC are critical for its diagnosis and treatment.

GRB14 is an adapter protein crucial in several cellular processes [34]. It belongs to the family of proteins connected with growth factor receptor-bound proteins, involved in signal transduction pathways [35]. GRB14 interacts with many signaling molecules and modulates diverse intracellular pathways. GRB14’s significance extends beyond metabolic and growth factor pathways [36]. Evidently, GRB14 contributes to various diseases, including cancer [8]. It influences cellular mechanisms related to malignancy progression, such as cell migration, invasion, and apoptosis [4]. GRB14’s interactions with key signaling molecules hint at its potential as a treatment target in malignancy management.

The PI3K/AKT pathway is a fundamental signaling cascade vital in modulating cellular growth, proliferation, survival, and metabolic process [37,38]. This mechanism is frequently dysregulated in cancer, contributing to tumor initiation, progression, and resistance to therapy [39]. The significance of PI3K/AKT pathway in malignancy renders it a promising therapeutic target. Researchers and pharmaceutical companies are actively developing drugs that target various components of the pathway, aiming to disrupt its aberrant signaling and halt tumor growth. However, the complexity of the pathway and its crosstalk with other signaling cascades challenges the development of effective therapies. Reportedly, the PI3K/AKT pathway is important in GC progression [40].

The COBL family, involved in morphogenesis, exerts regulatory control over neuronal actin networks, assuming a pivotal role in the process [11]. The COBL protein-like 1, encoded by the COBLL1 gene, is associated with genetic liability to metabolic disorders and specific types of cancer. The genetic locus COBLL1 is related to the pathogenesis of metabolic diseases and cortical surface alterations [41,42]. Clinically, heightened expression levels of COBLL1 demonstrated a significant correlation with leukemia [10]. The COBLL1 involvement in the NF-κB pathway of leukemia cells may be attributed to its capacity to enhance IKKγ stability [10].

This study accumulated evidence supporting the carcinogenic role of GRB14 in GC. Bioinformatics analysis revealed an elevated presence of GRB14 in human GC tissue compared to healthy tissue. The heightened GRB14 expression in GC relates to shorter survival times and poorer prognosis, indicating its potential to promote GC progression. Our *in vitro* experiments involving BGC-823 cells were conducted to investigate GC progression. The data demonstrated that downregulation of GRB14 curbs cell growth, invasion, cell cycle, and apoptosis in GC cell lines, whereas GRB14 overexpression yields contrary outcomes. These findings are consistent with the cancer-promoting effect exerted by GRB14 in numerous other prevalent tumors, such as hepatocellular carcinoma, glioblastoma, and thyroid cancer [4,8,43]. This study identified a novel interaction between GRB14 and COBLL1, resulting in the PI3K/AKT pathway activation via COBLL1 activation. Our findings suggested that GRB14 can modulate the GC cells’ growth and apoptosis by mediating the PI3K/AKT signaling pathway. These results are consistent with other studies suggesting that GRB14 promotes thyroid cancer progression through AKT phosphorylation [43]. Concludingly, our data strongly indicated GRB14 as an oncogene, holding promise as a predictive marker and treatment target in GC.
